# Chronic stress induces steatohepatitis while decreases visceral fat mass in mice

**DOI:** 10.1186/1471-230X-14-106

**Published:** 2014-06-10

**Authors:** Yun-Zi Liu, Ji-Kuai Chen, Yi Zhang, Xia Wang, Shen Qu, Chun-Lei Jiang

**Affiliations:** 1 Laboratory of Stress Medicine, Faculty of Psychology and Mental Health, Second Military Medical University, Shanghai, PR of China; 2 Department of Nautical Medicine, Second Military Medical University, Shanghai, PR of China; 3 Department of Pharmacology, School of Pharmacy, Second Military Medical University, Shanghai, PR of China; 4 Department of Endocrinology and Metabolism, Shanghai Tenth People’s Hospital, Tongji University School of Medicine, Shanghai, PR of China

**Keywords:** Chronic stress, Homeostasis, Steatosis, Inflammation, Visceral fat

## Abstract

**Background:**

Prolonged stress leads over time to allostatic load on the body and is likely to exacerbate a disease process. Long-term of stress exposure is one of a risk factor for metabolism-related diseases such as obesity and type 2 diabetes. However, the relationship between chronic stress and non-alcoholic fatty liver disease (NAFLD) remain unknown.

**Methods:**

To address the hypothesis that chronic stress associate to NAFLD development, we subjected C57bl/6 mice to electric foot shock and restraint stress for 12 weeks to set up chronic stress model. Then the serum and hepatic triglyceride (TG), total cholesterol (TC) were measured. Hepatic HE and Oil red O staining were used to specify the state of the NAFLD. To investigate whether inflammation takes part in the stress-induced NAFLD process, related visceral fat, serum and hepatic inflammatory factors were measured.

**Results:**

We observed that chronic stress led to an overall increase of hepatic triglyceride and cholesterol while decreasing body weight and visceral fat mass. Microvesicular steatosis, lobular inflammation and ballooning degeneration were seen in stress liver section. This effect was correlated with elevated hepatic and serum inflammatory factors. Although the amount of visceral fat was decreased in stress group, various adipocytokines were elevated.

**Conclusions:**

We showed that chronic stress is associated to NAFLD and chronic inflammation in visceral fat, though food intake and visceral fat mass were decreased. These results may contribute to better understanding of the mechanism from steatosis to steatohepatitis, and propose a novel insight into the prevention and treatment of NAFLD.

## Background

Non-alcoholic fatty liver disease (NAFLD), characterized by the accumulation of large droplets of triglyceride within hepatocytes in the absence of chronic alcohol consumption, is a leading cause of hepatic dysfunction. NAFLD represents a wide spectrum of diseases, ranging from simple steatosis, through steatosis with inflammation (non-alcoholic steatohepatitis, NASH) to cirrhosis. Although simple hepatic steatosis is a slowly-developed and asymptomatic disease, the next stage NASH is more likely to cause progressive cirrhosis, hepatocellular carcinoma and increased mortality [1]. The prevalence of NAFLD has increased dramatically over the last three decades, at nearly 15-30% in the general population in western countries and approximate 15% in big cities of China [2,3]. Despite its high prevalence, factors leading to progression from NAFLD to NASH remain obscure and there is no pharmacological agent approved for the treatment of NAFLD [4].

One major concern is chronic inflammation plays a key role in the pathogenesis of NAFLD [5]. IL-6 and TNF-α, two important inflammatory cytokines, profoundly increased in human patients with NAFLD [6]. Other studies also found a clear correlation between liver IL-6 and TNF-α expression level and NAFLD disease severity [7,8]. The absence of either IL-6 or TNF receptor 1 (TNFR1), decreased high-fat diet induced liver lipid accumulation and liver inflammation as assessed by reduced infiltration with macrophages and neutrophils [9].

Stress systems play a crucial role in maintaining homoeostasis to adapt to the environmental demands imposed by change [10]. However, strong and long-lasting stress stimuli could disturbances in the internal environment, thereby increasing the risk of various health problems. In modern society, which is characterized by a rapid pace of life, individuals are continuously confronted with an increasing number of stressful stimuli, such as emotional stimuli and social stress. It had been validated by clinical trials that chronic stress from work and low quality of life may be the risk factor of obesity and metabolism syndrome [11,12].

Since chronic stress could lead to systematic inflammation [13], we assume that chronic stress may take part in the process of NAFLD. Yet the relationship between chronic stress and NAFLD has not been clarified. Thus, the overall objective of this study was to demonstrate whether chronic stress could lead to NAFLD.

## Methods

### Experimental animals

Twenty female C57bl/6 mice (8 weeks of age; Sino-British SIPPR/BK Lad Animal Ltd, Shanghai, China) were allowed to acclimate to the environment for 7 days before the experiments began. Then they were randomly assigned to the control or stress group (n = 10, respectively). Control mice were left undistributed, while stress mice were suffered from chronic stress for 12 weeks. The detail process was described in next paragraph. Food intake was measured by weighing the pellets once per week. All animals were maintained on a 12-hour light/12-hour dark cycle with food and water freely available. The temperature of the colony room was maintained at 22°C to 23°C. Animal protocols were approved by the Animal Care and Use Committee of the Second Military Medical University.

### Stress protocol

During the 12 weeks, the mice in stress group were administered electric foot shock and restraint stress every day [14,15]. At 10:00 am, each mouse was placed in a box with a floor composed of stainless grids, and a scrambled electric shock was delivered through the floor grids by a PST-001 AC stimulator (StarMedical, Tokyo, Japan). An interval timer was connected to the stimulator to allow shocks for 7.5-second periods every 2 minutes, the intensity of which was 25v. At 7:00 pm, stressed mice were individually subjected to 2 h of restraint stress as described previously [16]. In brief, restraint stress was applied using a 50-mL conical centrifuge tube with multiple punctures that allowed for a close fit to mice. Control mice were kept isolated from stressed animals and maintained with access to food or water during the same period of stress process to avoid any acoustic or olfactory communication between the groups.

### Experimental procedures

After 12 weeks, all mice were fasted overnight then anesthetized using isoflurane. Blood was collected by orbital puncture, followed by sacrifice via cervical dislocation. Then the whole liver and intra-abdominal adipose were removed and weighed. For histology, a small part of liver and visceral fat were immediately fixed in 10% formalin, and processed for paraffin embedding. The rest of the liver was stored at −80°C for biochemical analysis.

### Determination of fat cell size

For these studies in fat tissue derived from mice euthanized at 12 week, two sections apart were selected, and the adipocyte cell size in three random fields in each section was determined. Because the cells were not uniformly round, the shortest length between the opposite cell membranes was measured routinely. For each cell, the inter-membrane distance was measured by including the central point in the cell in these planar images, where the lengths were measured in all of the complete cells within the field using Image J. Results were collated for each animal grouping.

### Hematoxylin-Eosin (HE) and oil red o staining of liver sections

Following fixation of the livers with10% formalin/phosphate-buffered saline, livers were sliced and stained with HE for histological examination. Liver steatosis was graded semi-quantitatively based on the percentage of hepatocytes according to the following criteria: grade 0, no hepatocytes involved; grade 1, 1% to 25% of hepatocytes involved; grade 2, 26% to 50% of hepatocytes involved; grade 3, 51% to 75% of hepatocytes involved; and grade 4, 76% to 100% of hepatocytes involved. Hepatic lipid content was also determined by staining with Oil Red O (Sigma). The percentage of the area occupied by oil red O-stained lipid droplets was calculated using Image J, averaging 3–5 separate, randomly selected 40× fields.

### Serum biochemical analysis

The serum triglyceride (TG), total cholesterol (TC), free fatty acid (FFA), alanine aminotransferase (ALT), and aspartate aminotransferase (AST) were measured by an automatic biochemistry analyzer(Hitachi 7170, Tokyo, Japan).

### Measurement of hepatic lipids

Livers were homogenized at 4°C in lysis buffer containing 50 mmol/L Tris (pH8.0), 150 mmol/L NaCl, 1% Triton X-100, and 0.5% sodium deoxycholate. Lipids from the total liver homogenate were extracted using the chloroform/methanol method (2:1), evaporated, and dissolved in 2-propanol. Amounts of TC and TG were measured by an automatic biochemistry analyzer (Hitachi 7170, Tokyo, Japan).

### Measurement of cytokines

Serum, adipose and liver samples were profiled with The Bio-Plex mouse Cytokine 17-Plex panel was used with the Bio-Plex Suspension Array System (Bio-Rad, Hercules, CA, USA) to profile expression of 7 inflammatory mediators, including IL-6, TNF-α, IL-1β, IL-18, MCP-1, MIP-2 and PAI-1. The assay was performed according to the manufacturer’s instructions.

### Data analysis

The data are presented as means ± SEM. Group means were compared utilizing a paired Student’s t-test or the Student-Newman-Keuls test when appropriate. The analysis of food intake and body weight was performed by ANOVA for repeated measures as appropriate. *P* < 0.05 was considered statistically significant.

## Results

### Chronic stress leads to decreased food intake and body weight gain

**Figure 1:**
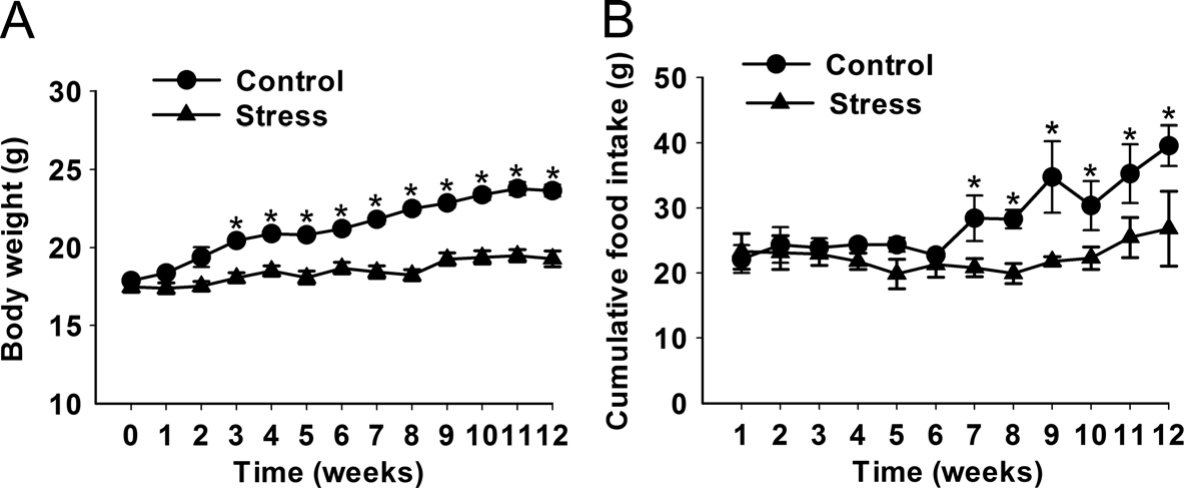
**Effects of stress on weight gain and food intake.** C57 BL/6 mice were received stress procedure for 12 weeks. **(A)** Changes in body weight. White squares = control (n = 10), Black squares = stress (n = 10). **(B)** Food intake during the course of the stress stimulating. Error bars represent standard error (SE). *P < 0.05, **P < 0.01 versus control mice.

To investigate whether chronic stress is linked to NAFLD progression, we made a 12 weeks stress protocol for C57BL/6 mice in order to lead to chronic stress status. During 12 weeks, the food intake and body weight of control group were steadily increased, while stress group didn’t show increased food intake and body weight gain. At the end of the time, both body weight and food intake gain were significantly lower in stress group (t (18) = 6.643, p <0.0001; t (18) = 3.882, p = 0.001) (Figure 1A, B).

### Chronic stress decreases visceral fat mass but increases inflammatory cytokines secretion

**Figure 2:**
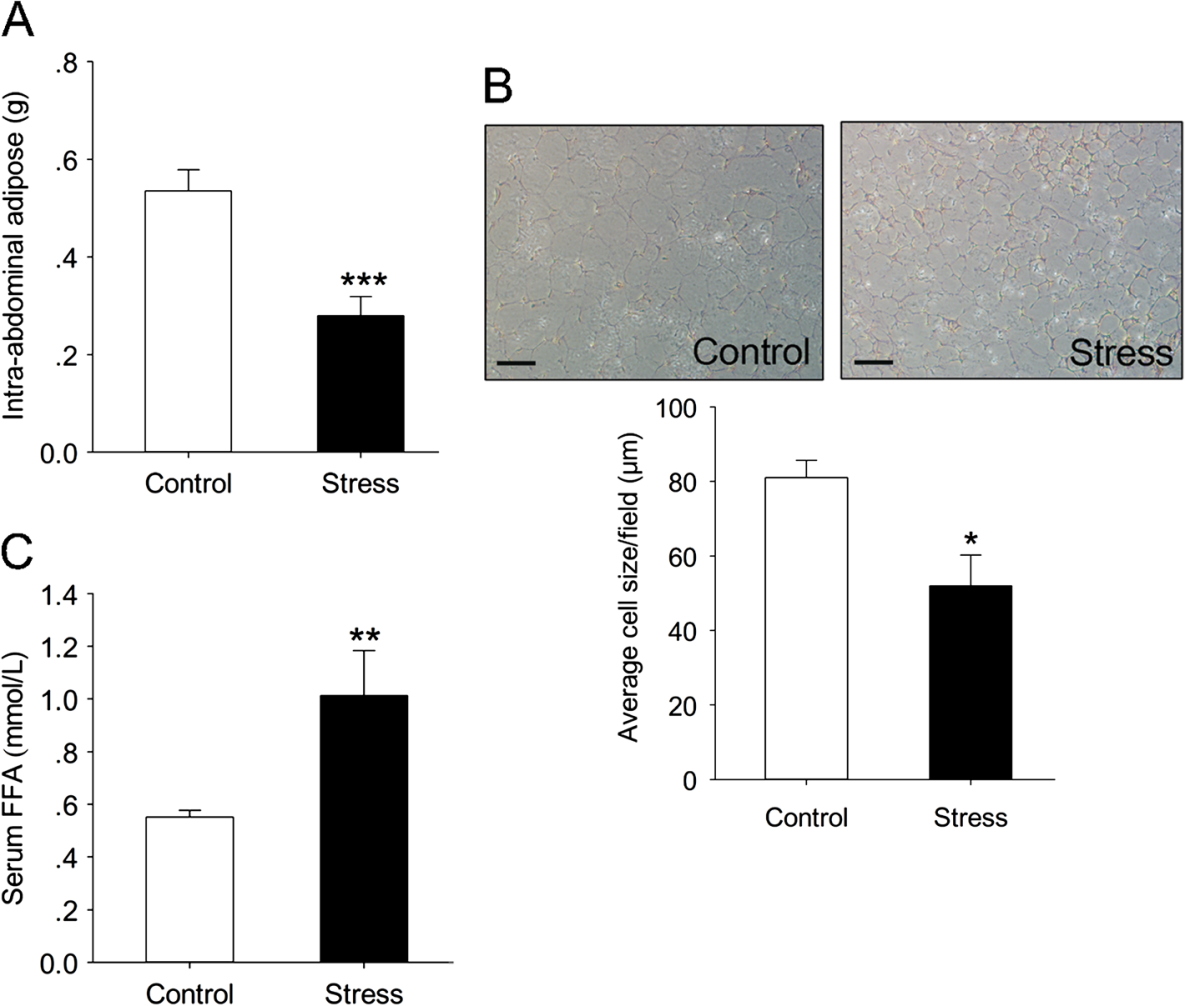
**Characterization of intra-abdominal adipose tissue and serum FFA of chronic stress mice. (A)** Weight of intra-abdominal adipose tissues from the control and stress mice received 12 weeks stress protocol. Error bars represent standard error (SE) (n = 10). **(B)** HE staining of intra-abdominal adipose tissue of stress mice received 12 weeks stress protocol. Magnification, ×100. Scale bars: 50 μm. Average cell sizes were calculated by Image J **(C)** Serum FFA content. Error bars represent standard error (SE). **P < 0.01, ***P < 0.001 versus control mice.

The amount of visceral adipose and adipocytokines is known to have a closely relationship with the development of NAFLD. Thus, we firstly measured visceral fat mass and serum FFA. The stress mice exhibited remarkable reduction of visceral mass compared to control ones (t (18) = 4.97, p = 0.0001) (Figure 2A), and HE staining showed that adipocytes of stress group were significantly smaller than control ones (t (8) = 3.055, p = 0.0157) (Figure 2B). Consistence with these results, Serum FFA was significantly elevated in stress group (t (18) = 2.846, p = 0.0107) (Figure 2C). These data indicate that chronic stress could lead to decreased amount of visceral fat and increased lipolysis.

**Figure 3:**
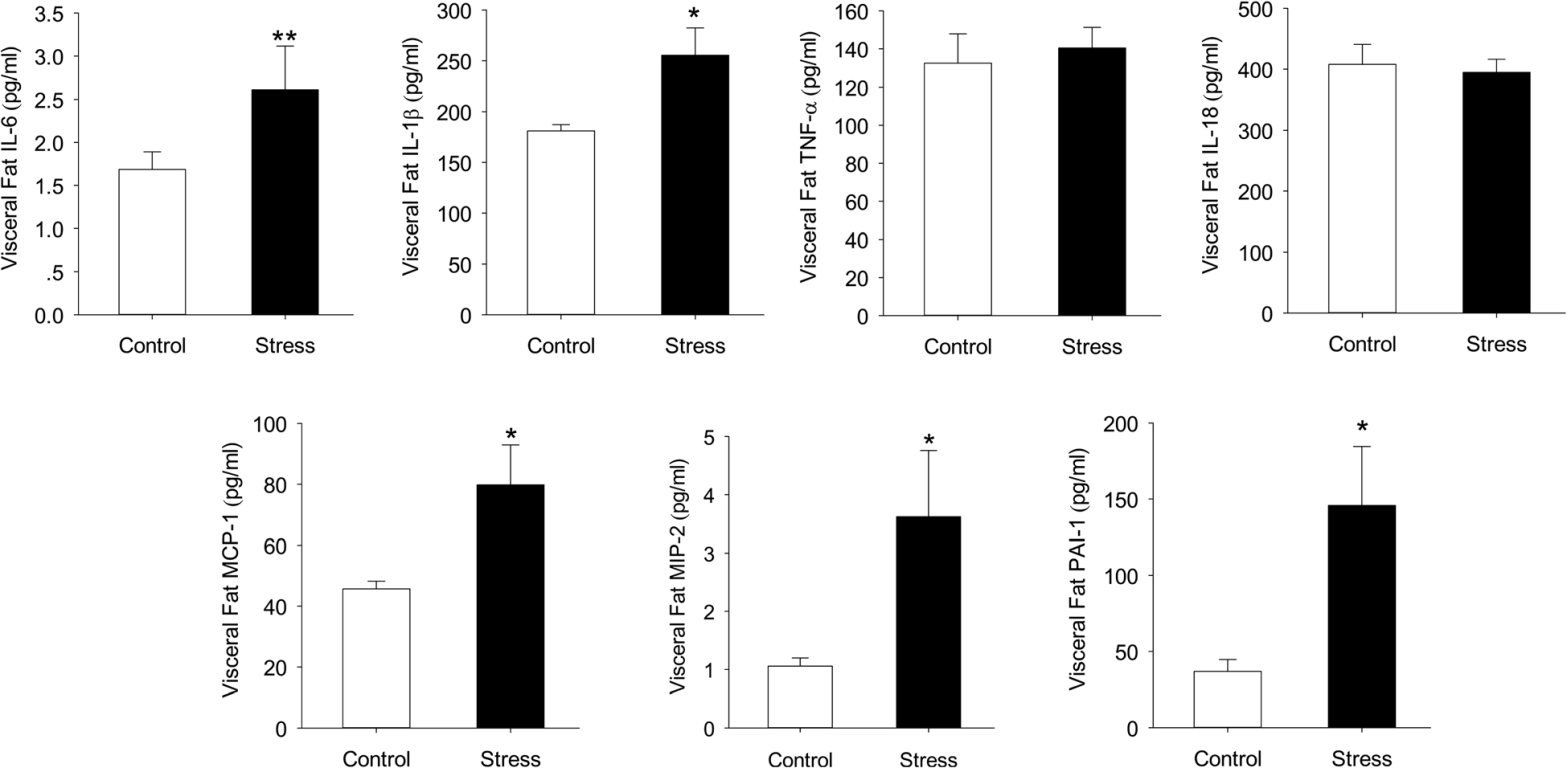
**Effects of chronic stress on the release of selected adipokines and cytokines by visceral adipose tissue from C57 BL/6 mice.** IL: interleukin, TNF-α: tumor necrosis factor-alpha, MCP-1: monocyte chemoattractant protein-1, MIP-2: macrophage inflammatory protein 2, PAI-1: plasminogen activator inhibitor-1. Error bars represent standard error (SE). *P < 0.05 versus control mice.

Since adipokine signaling is thought to be the means of NAFLD [17,18], we thought to determine how these molecules differed with chronic stress pattern. Adipose adipokine levels were examined after 12 weeks stress protocol. The results turned out that although the amount of visceral adipose was decreased, a variety of inflammatory factors of visceral adipose were elevated in stress mice. IL-6 and IL-1β were significantly increased (t (18) = 2.800, p = 0.011; t (18) = 2.712, p = 0.014), while others like TNF-α, IL-18 were not changed (t (18) = 0.670, p = 0.508; t (18) = 0.531, p = 0.600). Combined with cytokines, a lot of chemokines such as macrophage inflammatory protein 2 (MIP-2), monocyte chemoattractant protein-1(MCP-1) and PAI-1, related to inflammatory cells gathering were elevated too (t (18) = 2.236, p = 0.049; t (18) = 2.556, p = 0.029; t (18) = 2.756; p = 0.004) (Figure 3).

### Chronic stress induces steatosis

**Figure 4:**
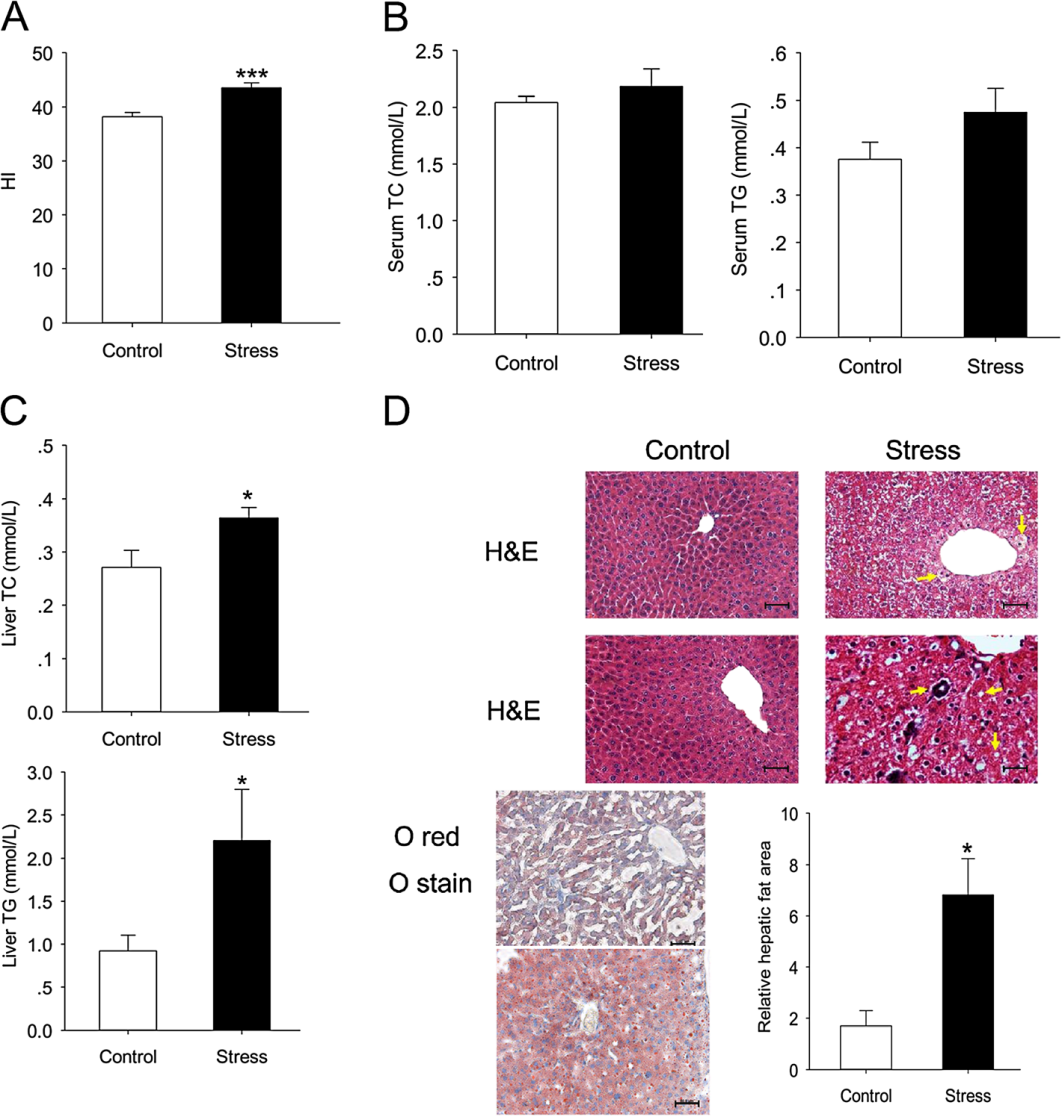
**Chronic stress induces hepatic TG and TC accumulation.** After mice were anesthetized, whole liver were removed and weighed. Hepatic lipids were extracted, and TG, TC concentrations were measured. **(A)** Hepatic index = Liver weight (mg)/body weight (g). **(B)** The levels of serum TC and TG content are represented in bar chart, respectively. **(C)** Liver TG and TC concentrations in stress group and control group. **(D)** Representative slides show hematoxylin and eosin (H&E)-stained and Oil red O-stained liver sections from control mice or stress mice for 12 weeks. The percentage of the area occupied by oil red O–stained lipid droplets was calculated using Image J, averaging 3–5 separate, randomly selected 40× fields. HE sections show chronic stress causing hepatocytes ballooning (yellow arrows). In addition, there are foci of inflammatory cell infiltration and lipid deposits (white arrows) Original magnification, ×100. Scale bars: 50 μm. Error bars represent standard error (SE). *P < 0.05, ***P < 0.001 versus control mice.

Although stress group had less food intake and weight gain, their hepatic index (HI, the ratio of liver weight to body weight × 100), which usually reflect the fatty liver status, was significantly elevated (t (18) = 0.588, p < 0.001) (Figure 4A). To ensure this phenomenon, Serum lipid metabolism indicators and liver steatosis indexes were evaluated. Serum TC and TG showed increasing tendency but had no statistical significance (t (11) = 1.560, p = 0.147; t (11) = 1.650, p = 0.127) (Figure 4B).Then we evaluated the liver TG and TC concentration and Oil red O staining to certify whether stress cause lipid deposition in liver. Quantitative analysis showed a significant increase in hepatic TG and TC content in stressed mice (t (18) = 2.770, p = 0.013; t (18) = 2.389, p = 0.028) (Figure 4C), which was confirmed by histological staining using Oil red O (t (8) =3.093, p = 0.015) (Figure 4D). Together, these data suggest that chronic stress could induce liver steatosis independent of the dietary factor.

### Stress not only leads to steatosis, but also NASH

**Figure 5:**
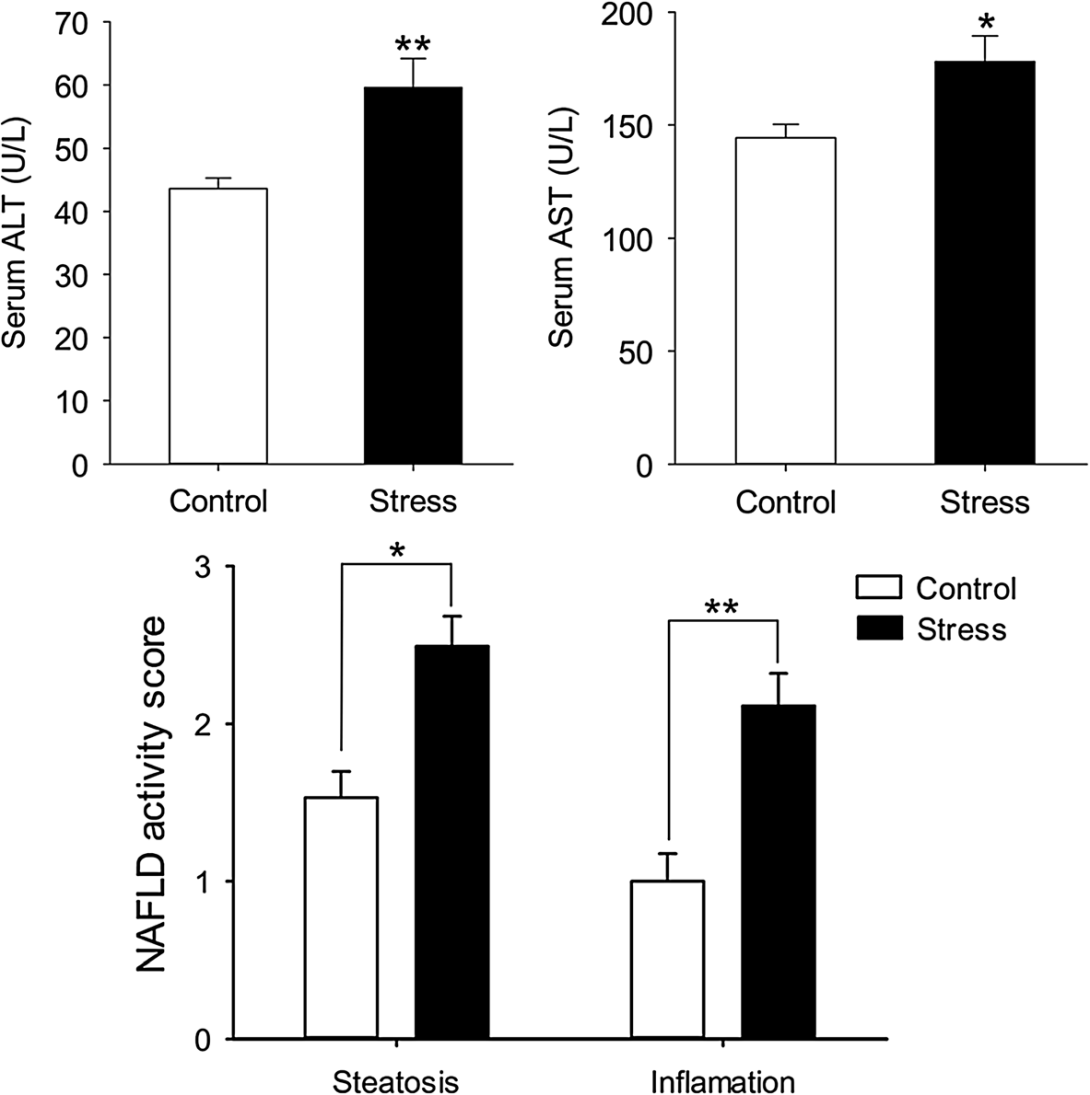
**The serum activities of two hepatic injury-associated enzymes, alanine aminotransferase (ALT), and aspartate aminotransferase (AST), at the end of the treatment period.** The NAFLD activity score is evaluated by New York NAFLD score system. *P < 0.05, **P < 0.01 versus control mice.

**Figure 6:**
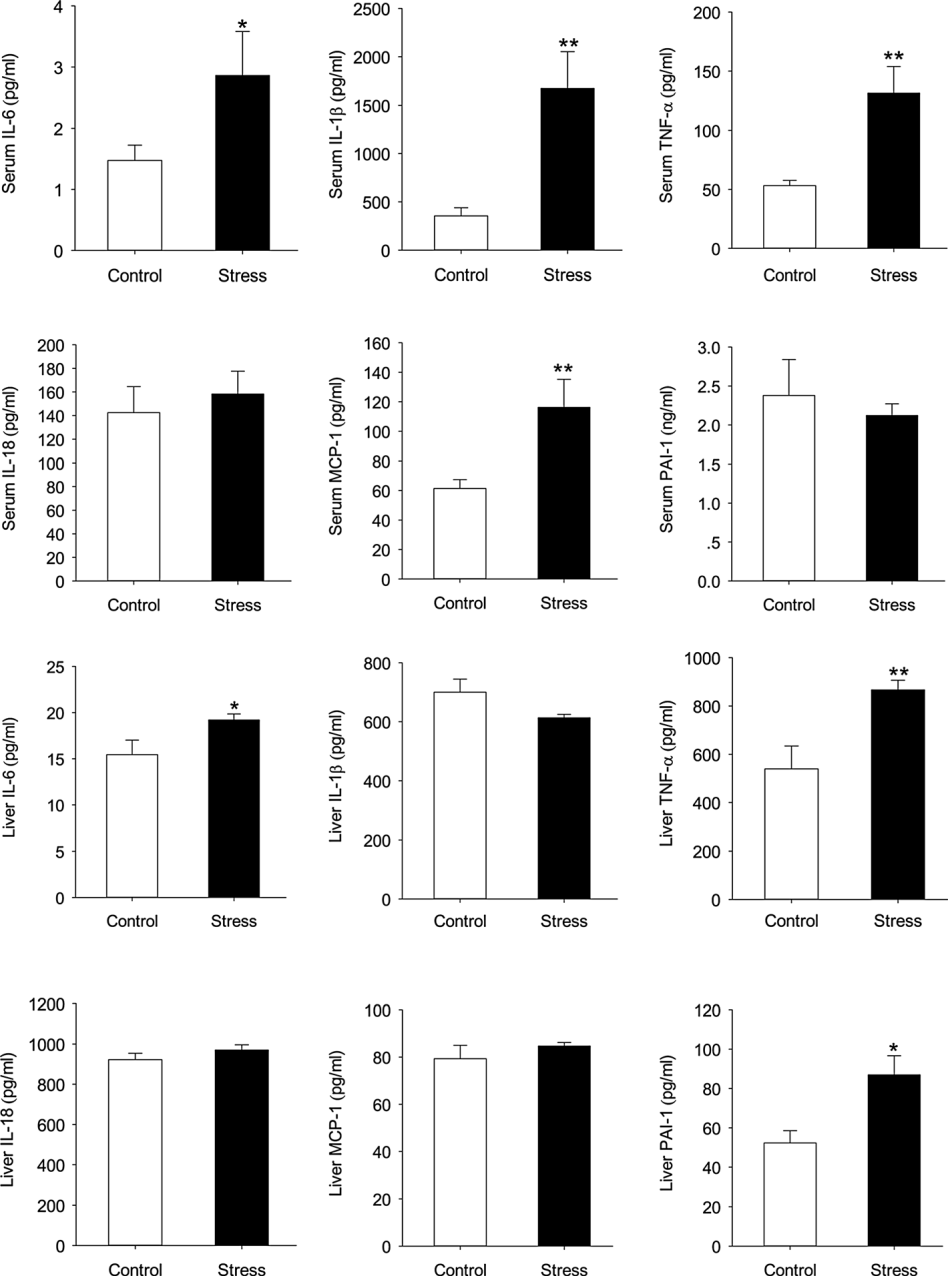
**Effects of chronic stress on the release of selected adipokines and cytokines by serum and liver from C57 BL/6 mice.** IL: interleukin, TNF-α: tumor necrosis factor-alpha, MCP-1: monocyte chemoattractant protein-1, PAI-1: plasminogen activator inhibitor-1. Error bars represent standard error (SE). *P < 0.05 versus control mice.

The former results illustrated that stress could induce liver steatosis and chronic inflammation state. We wanted to further investigate whether the mice encountered stress could develop to NASH. The results showed that stressed mice developed exacerbated NASH compared to wild-type mice as judged by increased levels of serum ALT and AST (t (13) = 3.271, p = 0.006; t (11) = 2.735, p = 0.0194), and NAFLD activity inflammation scores (t (18) = 2.742, p = 0.0134; t (18) = 3.899, p = 0.001) (Figure 5).To figure out which cytokines take part in NASH, we detected the cytokines closely related to NASH in both liver and serum. For IL-6 and TNF-α, stress mice had both higher serum and liver levels than control ones (serum IL-6: t (18) = 2.692, p = 0.0149; liver IL-6: t (18) = 2.784, p = 0.0123; serum TNF-α: t (9) = 3.479, p = 0.007; liver TNF-α: t (18) =3.023, p = 0.007). For IL-1β and MCP-1, serum levels were higher in mice given chronic stress compared with controls (t (18) = 3.365, p = 0.004; t (18) = 2.191, p = 0.042) while liver levels didn’t change (t (18) = 1.784, p = 0.091; t (18) =1.492, p = 0.153). For IL-18, the two groups showed same level in serum and liver after the stress treatment (t (18) = 1.334, p = 0.199; t (18) = 1.140, p = 0.269) (Figure 6).

## Discussion

The key finding in the present study is that mice could develop NASH only in intervention of chronic stress, despite the reduction in food intake and visceral fat, which always recognized as the risk factor of the metabolic disorder [19]. Furthermore, chronic stress could lead to chronic inflammatory state, including high concentration of inflammatory factors, such as TNF-α and IL-6, both in the circulation and liver. While previous studies mostly concentrated on the effect of chronic stress on obesity or metabolic syndrome combined with the high-fat diet [20], this is, to our knowledge, the first study indicating that chronic stress is a contributing factor of NAFLD, since the dietary factor and other related factors could be excluded in this experiment.

In some clinical trials, the amount of visceral fat is highly correlated with liver steatosis and steatohepatitis [21-23]. Obese persons with excess visceral adipose tissue are at higher risk for NAFLD components than those whose fat is predominantly located in the subcutaneous part [24]. Furthermore, weight loss induced by bariatric surgery is suggested to be responsible for the benefits in liver function and steatosis [25]. However, we noticed that, although stressed mice had less visceral fat content, they still exhibited a significantly higher hepatic TG and serum FFA level while the serum TC and TG were unchanged. The volume of fat tissue depends on its content of triglyceride. Therefore, we speculate that increase FFA was caused by lipotropic action in visceral adipose.

Apart from its ability of releasing and delivering FFA into the portal vein, visceral fat could secrete a variety of soluble factors which might be important sources to promote steatosis [26]. Then we detected the inflammation factors in visceral fat. The result showed that a variety of inflammatory cytokines and chemokines, such as IL-6, IL-1β, MIP-2 and MCP-1, elevated in the visceral adipose of stressed group. MIP-2 is a potent chemotactic agent for polymorphonuclear leukocytes, which was major secreted from stimulated macrophages [27]. MCP-1is secreted by a variety of cells as a response to several inflammatory stimuli, and play an important role in the stimulation of the inflammatory infiltrate. It could enhance expression of adhesion molecules in monocytes and promotion of pro-inflammatory cytokine synthesis [28,29]. All of these chemokines have the ability to activate and attract inflammatory cells, thus amplifying the inflammatory cascade. On the basis of these results, we infer that, chronic stress might induce steatosis through two ways: (1) chronic stress promotes visceral adipose lipolysis, thus increasing liver FFA input; (2) chronic stress lead to secretion of inflammatory cytokines and chemokines in visceral adipose tissue. However, the link between inflammatory adipose tissue and steatosis need to be further illustrated.

## Conclusions

In conclusion, this study provides a new insight into the pathogenesis of NAFLD. Our findings demonstrate that chronic stress status may be may be an important risk factor of NAFLD, although stressed mice had less food intake and weight gain. Furthermore, visceral adipose and hepatic inflammation may be the crucial mechanism of the stress-induced NAFLD, which need to be further proved. Up to now, there is a common sense that NAFLD is largely a hepatic manifestation of obesity and metabolic syndrome. Therefore, these results may remind us that, those who have normal weight and hip-waist ratio but suffer long-term stress may also have the chance to have NAFLD. To prevent NAFLD, not only should we restrict the calorie properly, we also should concentrate on stress management.

## Competing interests

The authors disclose no potential competing of interest.

## Authors’ contributions

YZL designed and executed the experiments, interpreted data, and wrote the manuscript. JKC, YZ and XW performed molecular biology experiment and animal experiment. SQ and CLJ conceived the study, and participated in its design and helped to draft the manuscript. All authors read and approved the final manuscript.

## Pre-publication history

The pre-publication history for this paper can be accessed here:

http://www.biomedcentral.com/1471-230X/14/106/prepub

## References

[B1] Adams LA Lymp JF St SJ Sanderson SO Lindor KD Feldstein A Angulo P The natural history of nonalcoholic fatty liver disease: a population-based cohort study Gastroenterology 2005 129 113 121 16012941 10.1053/j.gastro.2005.04.014

[B2] Ratziu V Bellentani S Cortez-Pinto H Day C Marchesini G A position statement on NAFLD/NASH based on the EASL 2009 special conference J Hepatol 2010 53 372 384 20494470 10.1016/j.jhep.2010.04.008

[B3] Fan JG Farrell GC Epidemiology of non-alcoholic fatty liver disease in China J Hepatol 2009 50 204 210 19014878 10.1016/j.jhep.2008.10.010

[B4] Lomonaco R Sunny NE Bril F Cusi K Nonalcoholic fatty liver disease: current issues and novel treatment approaches Drugs 2013 73 1 14 23329465 10.1007/s40265-012-0004-0

[B5] Hotamisligil GS Inflammation and metabolic disorders Nature 2006 444 860 867 17167474 10.1038/nature05485

[B6] Crespo J Cayon A Fernandez-Gil P Hernandez-Guerra M Mayorga M Dominguez-Diez A Fernandez-Escalante JC Pons-Romero F Gene expression of tumor necrosis factor alpha and TNF-receptors, p55 and p75, in nonalcoholic steatohepatitis patients Hepatology 2001 34 1158 1163 11732005 10.1053/jhep.2001.29628

[B7] Abiru S Migita K Maeda Y Daikoku M Ito M Ohata K Nagaoka S Matsumoto T Takii Y Kusumoto K Nakamura M Komori A Yano K Yatsuhashi H Eguchi K Ishibashi H Serum cytokine and soluble cytokine receptor levels in patients with non-alcoholic steatohepatitis Liver Int 2006 26 39 45 16420507 10.1111/j.1478-3231.2005.01191.x

[B8] Manco M Marcellini M Giannone G Nobili V Correlation of serum TNF-alpha levels and histologic liver injury scores in pediatric nonalcoholic fatty liver disease Am J Clin Pathol 2007 127 954 960 17509993 10.1309/6VJ4DWGYDU0XYJ8Q

[B9] Park EJ Lee JH Yu GY He G Ali SR Holzer RG Osterreicher CH Takahashi H Karin M Dietary and genetic obesity promote liver inflammation and tumorigenesis by enhancing IL-6 and TNF expression Cell 2010 140 197 208 20141834 10.1016/j.cell.2009.12.052 PMC2836922

[B10] Chrousos GP Stressors, stress, and neuroendocrine integration of the adaptive response. The, Hans Selye Memorial Lecture Ann N Y Acad Sci 1997 1998 851 311 335 10.1111/j.1749-6632.1998.tb09006.x 9668623

[B11] Chandola T Brunner E Marmot M Chronic stress at work and the metabolic syndrome: prospective study BMJ 2006 332 521 525 16428252 10.1136/bmj.38693.435301.80 PMC1388129

[B12] Moles A Bartolomucci A Garbugino L Conti R Caprioli A Coccurello R Rizzi R Ciani B D'Amato FR Psychosocial stress affects energy balance in mice: modulation by social status Psychoneuroendocrinology 2006 31 623 633 16616814 10.1016/j.psyneuen.2006.01.004

[B13] Marchesini G Bugianesi E Forlani G Cerrelli F Lenzi M Manini R Natale S Vanni E Villanova N Melchionda N Rizzetto M Nonalcoholic fatty liver, steatohepatitis, and the metabolic syndrome Hepatology 2003 37 917 923 12668987 10.1053/jhep.2003.50161

[B14] Knepel W Nutto D Hertting G Evidence for inhibition by beta-endorphin of vasopressin release during foot shock-induced stress in the rat Neuroendocrinology 1982 34 353 356 6281677 10.1159/000123327

[B15] Blanc G Herve D Simon H Lisoprawski A Glowinski J Tassin JP Response to stress of mesocortico-frontal dopaminergic neurones in rats after long-term isolation Nature 1980 284 265 267 7189015 10.1038/284265a0

[B16] Yamamoto K Takeshita K Shimokawa T Yi H Isobe K Loskutoff DJ Saito H Plasminogen activator inhibitor-1 is a major stress-regulated gene: implications for stress-induced thrombosis in aged individuals Proc Natl Acad Sci U S A 2002 99 890 895 11792849 10.1073/pnas.022608799 PMC117401

[B17] Jarrar MH Baranova A Collantes R Ranard B Stepanova M Bennett C Fang Y Elariny H Goodman Z Chandhoke V Younossi ZM Adipokines and cytokines in non-alcoholic fatty liver disease Aliment Pharmacol Ther 2008 27 412 421 18081738 10.1111/j.1365-2036.2007.03586.x

[B18] Tilg H Hotamisligil GS Nonalcoholic fatty liver disease: cytokine-adipokine interplay and regulation of insulin resistance Gastroenterology 2006 131 934 945 16952562 10.1053/j.gastro.2006.05.054

[B19] Bergman RN Kim SP Catalano KJ Hsu IR Chiu JD Kabir M Hucking K Ader M Why visceral fat is bad: mechanisms of the metabolic syndrome Obesity (Silver Spring) 2006 14 Suppl 1 16S 19S 16642958 10.1038/oby.2006.277

[B20] Czech B Neumann ID Muller M Reber SO Hellerbrand C Effect of chronic psychosocial stress on nonalcoholic steatohepatitis in mice Int J Clin Exp Pathol 2013 6 1585 1593 23923077 PMC3726974

[B21] Eguchi Y Eguchi T Mizuta T Ide Y Yasutake T Iwakiri R Hisatomi A Ozaki I Yamamoto K Kitajima Y Kawaguchi Y Kuroki S Ono N Visceral fat accumulation and insulin resistance are important factors in nonalcoholic fatty liver disease J Gastroenterol 2006 41 462 469 16799888 10.1007/s00535-006-1790-5

[B22] Angulo P NAFLD, obesity, and bariatric surgery Gastroenterology 2006 130 1848 1852 16697746 10.1053/j.gastro.2006.03.041

[B23] van der Poorten D Milner KL Hui J Hodge A Trenell MI Kench JG London R Peduto T Chisholm DJ George J Visceral fat: a key mediator of steatohepatitis in metabolic liver disease Hepatology 2008 48 449 457 18627003 10.1002/hep.22350

[B24] Hamdy O Porramatikul S Al-Ozairi E Metabolic obesity: the paradox between visceral and subcutaneous fat Curr Diabetes Rev 2006 2 367 373 18220642 10.2174/1573399810602040367

[B25] Engl J Sturm W Sandhofer A Kaser S Tschoner A Tatarczyk T Weiss H Tilg H Patsch JR Ebenbichler CF Effect of pronounced weight loss on visceral fat, liver steatosis and adiponectin isoforms Eur J Clin Invest 2008 38 238 244 18312420 10.1111/j.1365-2362.2008.01929.x

[B26] Choi S Diehl AM Role of inflammation in nonalcoholic steatohepatitis Curr Opin Gastroenterol 2005 21 702 707 16220049 10.1097/01.mog.0000182863.96421.47

[B27] Wolpe SD Sherry B Juers D Davatelis G Yurt RW Cerami A Identification and characterization of macrophage inflammatory protein 2 Proc Natl Acad Sci U S A 1989 86 612 616 2643119 10.1073/pnas.86.2.612 PMC286522

[B28] Jiang Y Beller DI Frendl G Graves DT Monocyte chemoattractant protein-1 regulates adhesion molecule expression and cytokine production in human monocytes J Immunol 1992 148 2423 2428 1348518

[B29] Devalaraja MN Mcclain CJ Barve S Vaddi K Hill DB Increased monocyte MCP-1 production in acute alcoholic hepatitis Cytokine 1999 11 875 881 10547276 10.1006/cyto.1999.0495

